# Intestinal tract is an important organ for lowering serum uric acid in rats

**DOI:** 10.1371/journal.pone.0190194

**Published:** 2017-12-21

**Authors:** Yu Yun, Hua Yin, Zhiyi Gao, Yue Li, Tao Gao, Jinlian Duan, Rong Yang, Xianxiang Dong, Lumei Zhang, Weigang Duan

**Affiliations:** 1 Kunming Key Laboratory of Molecular Biology for Sinomedicine, Faculty of Basic Medicine, Yunnan University of Traditional Chinese Medicine, Kunming, China; 2 School of Basic Medicine, Kunming Medical University, Kunming, China; Leibniz-Institut fur Pflanzengenetik und Kulturpflanzenforschung Gatersleben, GERMANY

## Abstract

The kidney was recognized as a dominant organ for uric acid excretion. The main aim of the study demonstrated intestinal tract was an even more important organ for serum uric acid (SUA) lowering. Sprague-Dawley rats were treated normally or with antibiotics, uric acid, adenine, or inosine of the same molar dose orally or intraperitoneally for 5 days. Rat’s intestinal tract was equally divided into 20 segments except the cecum. Uric acid in serum and intestinal segment juice was assayed. Total RNA in the initial intestinal tract and at the end ileum was extracted and sequenced. Protein expression of xanthine dehydrogenase (XDH) and urate oxidase (UOX) was tested by Western blot analysis. The effect of oral UOX in lowering SUA was investigated in model rats treated with adenine and an inhibitor of uric oxidase for 5 days. SUA in the normal rats was 20.93±6.98 μg/ml, and total uric acid in the intestinal juice was 308.27±16.37 μg, which is two times more than the total SUA. The uric acid was very low in stomach juice, and attained maximum in the juice of the first segment (duodenum) and then declined all the way till the intestinal end. The level of uric acid in the initial intestinal tissue was very high, where XDH and most of the proteins associated with bicarbonate secretion were up-regulated. In addition, SUA was decreased by oral UOX in model rats. The results suggested that intestinal juice was an important pool for uric acid, and intestinal tract was an important organ for SUA lowering. The uric acid distribution was associated with uric acid synthesis and secretion in the upper intestinal tract, and reclamation in the lower.

## Introduction

Uric acid is the final product of purine nucleoside metabolism by xanthine dehydrogenase (XDH, EC 1.17.1.4) in humans. Humans do not express urate oxidase (UOX, EC 1.7.3.3, uricase) that transforms uric acid to allantoin, a more soluble substance than uric acid [1,2]. The level of uric acid is well controlled mainly by the balance between production from purine nucleosides in liver and excretion into urine through kidneys [1,2]. Although its physiological role is poorly understood, due to its antioxidant activity, uric acid is thought to protect neuronal cells and also play a role in maintaining the blood pressure [3]. Uric acid is almost insoluble in water, and easily forms precipitates (tophi) in the peripheral and terminal tissues or organs like kidneys, joints, and ear lobes. Therefore, it is suggested that serum uric acid (SUA) levels should be kept below 7 mg/dL (about 420 μmol/L) to prevent hyperuricemia [4]. Increased SUA is an important clinical risk factor for gout, chronic kidney disease (CKD) [5,6,7,8], and some chronic cardiovascular diseases [9].

Kidney is accepted as the main way for excretion of about two thirds of the total uric acid [3]. The opinion was almost unshakable with a line of evidence. Several transporters in kidney for uric acid excretion (secretion) and re-uptake (reabsorption) were observed, which include ABCG2, MRP4, NPT1 OAT10, URAT1 and GLUT9 [1]. The mechanism of uricosuric agents like probenecid and benzbromarone involves inhibition of urate reabsorption in kidney, which is transduced by URAT1 and GLUT9 [10,11]. Since there were many studies supporting that the kidney is the most important organ for uric acid excretion, the above mentioned uricosuric agents were considered as the most frequent choice to lower SUA [12]. However, the uricosuric agents must be absorbed and carried to the renal tubules to inhibit the transporters associated with uric acid reabsoption, and may cause some systemic adverse reactions like liver and kidney damage [13]. Other therapeutics such as XDH inhibitors also share similar shortcomings [14]. According to some scientists, if endogenous uric acid was excreted through intestinal tract [15], then the drug treatment for hyperuricemia could be done locally, which in turn avoid the systemic adverse reactions.

According to the present data, about one-third of the total uric acid was excreted through intestinal tract. A few preclinical studies [16,17] proved that uric acid existed in the upper intestinal tract of mice. However, the total uric acid in the intestinal tract is not systemically evaluated, and the relationship of uric acid between the intesitinal tract (juice) and in blood (serum) is still not clear. Therefore, this study was conducted to prove that the intestinal tract is an important place for uric acid distribution, then an important organ for uric acid removal, enven more important than kidney.

## Materials and methods

### Materials

Male Sprague-Dawley (SD) rats aged 2 months and weighing 180–200 g, were obtained from Jianyang Dashuo Science and Technology Ltd., Chengdu, China (Certification No. SCXK (Chuan) 2008–24). Rats were housed at 22°C temperature, at 45–55% humidity-controlled conditions, and under natural light. This project was approved by the Experimental Animal Committee of Yunnan University of Traditional Chinese Medicine.

Uric acid was purchased from Tokyo Into Industrial Co., Ltd. (Tokyo, Japan). Adenine and potassium oxonate (a uricase inhibitor) was purchased from Shanghai Yuanye Biotech Ltd (Shanghai, China). Inosine was purchased from Sangon Biotech (Shanghai China). Standard solution of uric acid (1000 μg/ml, 5952 μM), uric acid assay kits of phosphotungstic acid method, protein assay kits of BCA (bicinchoninic acid) method, blood urea nitrogen (BUN) assay kits of diacetyl monoxime method, and blood creatinine (Cr) assay kits of picric acid method were purchased from Nanjing Jiancheng Bioengineering Institute (Nanjing, China). TRIzol Plus RNA Purification kit was purchased from Introgen (Carlsbad, CA, USA). Rabbit anti-UOX was purchased from Shanghai Ruiqi Biological Technology Co. Ltd (Shanghai, China). Rabbit anti- XDH was purchased from Abcam Trading (Shanghai) Company Ltd (Shanghai, China), and goat anti- rabbit antibody linked with HRP was purchased from Boster Biological Engineering Co., Ltd (Wuhan, China). UOX (4.41 u/mg) was purchased from Worthington Biochemical Corporation (Freehold, N.J., USA). Enhanced chemoluminescence (ECL) detection kits were purchased from Pierce Biotechnology Inc (Rockford, IL, USA). Ultra-pure water was obtained by Milli Q water purification system manufactured by EMD Millipore Group (Darmstadt, Germany). A pH meter with micro electrode (pH-30) was manufactured by Thermo Orion, USA. NanoDrop ND-1000 spectrophotometer was manufactured by PeqLab, Erlangen, Germany. The multimicroplate reader of Infinite 200pro was manufactured by Tecan Group (Mannedorf, Switzerland). Other instuments or reagents used in the present study were made in China.

### Animal treatment

In order to investigate the distribution of uric acid in different tissues in normal rats, SD rats were fasted for 36 h before sacrificing. Rats were intraperitoneally anaesthetized with urethane (1.0 g/kg). The abdomen of rat was opened, blood samples were drawn via the abdominal aorta, and organs including liver, spleen, lung, bladder, pancreas, kideny, testical, brain, heart, ectogluteus, duodenum (5cm), and the last 5 cm of ileum were harvested. The intestinal tract was opened and the inner wall was cleaned with a cotton swab, and was rinsed with 1 ml normal saline twice. The sample of organs or tissues was frozen at -40°C for use or homogenated on ice immediately.

In order to investigate uric acid in intestinal juice, SD rats were treated normally for 3 days and were randomized to different groups, namely, normal and treatment groups. Rats in normal group were served as control and treated with normal saline of the same volume. Animals in the treatment groups were intraperitoneally or intragastrically administrated with antibiotics (antibiotic treated group), adenine (400 mg/kg, adenine-po or ip group), sodium urate (400 mg/kg, urate-po or ip group), or inosine (680 mg/kg, inosine-po or ip group) for 5 days. For ease of collection of intestinal juice, all the rats were fasted for 36 h before sacrificing. When the last administration was taken for 2 h, rats were intraperitoneally anaesthetized with urethane (1.0 g/kg). The abdomen of rat was opened, blood samples were drawn via the abdominal aorta, then the stomach and the intestinal tract from duodenum to rectum were harvested. The stomach juice was directly collected. The intestinal tract was equally divided into 20 segments (about 5 cm per segment, segment 1 was duodenum) according to the length of the intestinal tract except cecum (about 10 cm). Both ends of the intestinal segment were clamped with tissue forceps to prevent intestinal juice losing and the intestinal juice in the segments were collected as soon as a segment was cut, and pH of the juice was tested with the pH meter with micro electrode. The inner wall of the intestinal segment was rinsed with normal saline of 200 μl, and the two parts of liquid sample were combined and used as intesitnal juice sample for assay. When the blood sample had coagulated, the serum sample was obtained by spinning at 3,000×g for 5 min at 4°C. All samples were kept at -40°C for testing or assayed immediately.

### Uric acid assay

The concentration of uric acid (μg/ml) in the serum samples, stomach juice, and the supernatant of tissue homogenate was assayed with uric acid assay kits according to the standard operation procedure (SOP) provided by the manufacturer. The protein in all the samples was assayed with protein assay kits.

### RNA sequencing

After harvesting the intestinal tract in the normal group, the tract was opened, the contents were removed, and the inner wall was cleaned with a cotton swab and rinsed with 1.0 ml normal saline twice. The tract was frozen with liquid nitrogen and ground to powder. The total RNA in the powder was extracted and purified by TRIzol Plus RNA Purification kit. RNA quantity and quality were measured by the NanoDrop ND-1000 spectrophotometer. RNA integrity was assessed by standard denaturing agarose gel electrophoresis [7,18].

Double-stranded cDNA (ds-cDNA) was synthesized from total RNA using an Invitrogen SuperScript ds-cDNA synthesis kit in the presence of 100 pmol/L oligo dT primers. The cDNA was sequenced by Sangon Biotech (Shanghai, China). Expected value of FPKM (fragments per kilobase of transcript sequence per million base pairs sequenced) was used for expression normalization [19, 20]. The analysis of the FPKM of a gene between different intestinal tissues was also made by Sangon Biotech.

### Bacterial abundance determined by 16S rRNA

After harvesting of the intestinal tract in another normal group, the intestinal tract was divided into similar segments. Segments 1–18 were frozen with liquid nitrogen and ground to powder. Total DNA in the powder of every segment was extracted and purified by an E.Z.N.ATM Mag-Bind Soil DNA Kit (Omega Biotec, Guangzhou, China). All the DNAs in the segments were dissolved in 70 μl buffer provided by the E.Z.N.ATM Mag-Bind Soil DNA Kit. DNA quantity and quality were measured by the NanoDrop ND-1000 spectrophotometer. The DNA coded by bacterial 16S rRNA was amplified using 25 cycles with the 16SV3-V4 barcode primers Bakt_341F (CCTACGGGNGGCWGCAG) and Bakt_805R (GACTACHVGGGTATCTAATCC) [21]. The 5’ end of the primers was designed as adapters for purification, which were CCCTACACGACGCTCTTCCGATCTG and GACTGGAGTTCCTTGGCACCCGAGAATTCCA, respectively [21]. The purified 16S rRNA was assessed by standard agarose gel electrophoresis. Bacterial abundance was judged by the abundance of 16S rRNA it contained.

### Western blot analysis

Rat intestinal tract (about 5 cm) was homogenated in icy isotonic lysis buffer (25 mmole/L Tris, pH 7.4, 150 mmole/L NaCl, complete protease inhibitors from Roche, 1 mmole/L sodium orthovanadate, 1 mmole/L sodium pyrophosphate, 10 mmole/L β-glycerophosphate) [22]. Supernatant for the analysis was obtained by spinning the homogenate at 5,000×g for 5 min at 4°C.

Proteins were detected by Western blot, which was as follows [23,24,25]. Protein (50.0 μg) of different samples were applied on to the sodium dodecyl sulfate-polyacrylamide gel electrophoresis (SDS-PAGE). The protein in SDS-PAGE was transferred to a polyvinylidene fluoride (PVDF) filter by an electrical current of 10 V for 60 min in a semi-dry electrophoretic transfer cell. The PVDF filter was blocked with 3% bovine serum albumin at an ambient temperature for 2 hr; then, bathed in the primary antibody solution at 4°C overnight. The filter was rinsed with TST Buffer (20 mmole/L Tris-HCl, pH 7.5, 0.05% Tween-20) for 10 min three times, and bathed in a solution of goat anti-rabbit antibody linked with HRP (1:400) for another 2 hr. The PVDF filter was rinsed with TST buffer for 10 min three times and developed by enhanced chemoluminescence (ECL) detection kits. The band brightness was quantified by ImageJ 1.48v software.

### SUA affected by oral UOX in rats

Male rats were randomized to model group, UOX group and normal group. Rats in model group were gavaged with Mixture A of 10 ml/kg, containing adenine of 15 mg/ml, potassium oxonate of 20 mg/ml, omeprazole of 0.4 mg/ml and CMC-Na of 0.5% in the morning and gavaged with Mixture B of 5 ml/kg, containing NaHCO_3_ of 12 mg/ml and 50% horse serum in the afternoon, to copy a hyperuricemia model [26]. Rats in UOX group were gavaged with Mixture A of same volume in the morning and gavaged with Mixture B of same volume but added UOX of 4 u/ml in the afternoon. Rats in normal group were gavaged with normal saline of the same volume both in the morning and the afternoon. Administration was taken for 5 days. The rats was intraperitoneally anaesthetized with urethane (1.0 g/kg), and blood was drawn to obtain serum sample. SUA, BUN, and Cr in serum was assayed with assay kits.

### Statistical analysis

The relative density of stomach juice and intestinal juice was assumed as “1”, and its volume was calculated from its weight. Total uric acid in rat serum was calculated by multiplying SUA with 1/26 of its body weight (the total blood is about 1/13 of the body weight, and the serum is about 1/2 of the blood) [27]. Total uric acid in rat intestinal juice was calculated by summing up its content in all the intestinal segment together. The IS ratio of uric acid between **i**ntestine and **s**erum was calculated by formula “IS Ratio = (Total in Intestinal Juice)/(Total in Serum)”.

Values were expressed as mean ± SD (standard deviation) or SE (standard error). ANOVA (analysis of variance) was performed to compare means between different groups. If there was a significance, post-hoc statistical tests between every two groups were performed. Statistical significance was accepted at P < 0.05.

## Results

### Distribution of uric acid in different tissues in normal rats

The distribution of uric acid in different tissues was showed in Table 1. The tissue with highest level of uric acid was duodenum, then ileum and liver, belonging to alimentary system, which suggested that the alimentary system was a dominant place for uric acid.

**Table 1:** Distribution of uric acid in male rat’s tissues (mean ± SD, n = 10).

Tissue	Uric acid in tissue (μg/g tissue)	Uric acid in tissue (μg/mg protein)	Uric acid in gastrointestinal juice (μg/ml)
duodenum	1290.09±316.57	18.02±4.50	221.50±36.00
ileum ^a^	704.96±242.01	12.80±3.61	30.55±1.70
liver	552.62±195.58	5.02±0.32	55.48±9.69 ^c^
spleen	540.72±171.06	7.89±2.40	
Colon	442.25±254.20	8.01±4.13	33.70±4.23
Lung	396.25±265.56	8.51±3.95	
bladder	247.55±70.88	6.77±1.18	
stomach	202.55±101.40	8.92±2.16	5.09±0.40
pancreas	179.15±31.95	2.12±0.13	
Kidney	174.51±19.61	2.81±0.04	
Testicle	94.81±16.64	1.91±0.49	
Brain	88.96±55.76	1.80±1.14	
Blood	80.76±8.70	0.08±0.01	
Heart	74.83±16.87	1.82±0.30	
skeletal muscle ^b^	45.98±12.79	1.30±0.34	
Serum	25.96±3.35	0.05±0.01	

^a^The last 5 cm of ileum.

^b^Ectogluteus.

^c^The values were from the bile of rabbit that fasted for 36 hr.

Comparatively, uric acid in serum was at the lowest level. Blood, testicle, brain, heart and skeletal muscle were the tissues with relative low levels of uric acid.

### Uric acid in intestinal juice of different groups

SUA in the normal group was about 20 μg/ml (Table 2), but uric acid in the blood was 85 μg/ml (84.80±9.13 μg/ml), which was about four times of SUA. These results suggested that there could be more uric acid inside the cells. The total uric acid in normal serum was about 150 μg per rat; while the total uric acid in intestinal juice was about 300 μg per rat, twice of that of the above (Table 2). And the similar results were also seen in other groups except adenine-ip group (Table 2). The results suggested that intestinal juice is a very important pool for uric acid distribution, even more important than serum.

**Table 2:** Uric acid in different groups (n = 10).

Groups	Reagents	Treatment for 5 days	SUA (μg/ml mean±SD)	a, Total SUA (μg, mean±SE)	b, Total UA in intestinal juice (μg, mean±SE)	IS ratio (b/a, mean±SE)
normal	none		20.93±6.98	152.02±5.82	308.27±16.37	2.30±0.16
Antibiotic treated	Gentamincin (50 μg/ml) + vancomycin (50 μg/ml)	Free drink	26.96±6.67	229.14±6.55	386.86±15.73	1.91±0.13
Urate-ip	Sodium urate 400mg/kg	ip.	27.65±8.26	217.37±8.88	376.55 ±24.41	1.69±0.06
Adenine-ip	Adenine 400mg/kg	ip.	25.45±3.81	169.23±3.44	541.63 ±24.15	3.27±0.12
Inosine-ip	Inosine 680 mg/kg	ip.	17.65±4.34	148.56±3.44	385.77±9.43	2.66±0.08
Adenine-po	Adenine 400mg/kg	po.	40.77±7.52*	337.65±6.26*	175.97±11.09*	0.56±0.05*
Inosine-po	Inosine 680 mg/kg	po.	33.16±6.17*	256.12±6.44*	349.46±13.33	1.45±0.05
Urate-po	Sodium urate 400mg/kg	Po.	26.82±6.78	206.2±5.63	-	-

SUA, serum uric acid; UA, uric acid.

*P<0.05 vs normal group, ANOVA; the molar dosage of 680 mg inosine is equal to that of 400 mg adenine.

Due to their low oral bioavailability, a free drink of gentamicin (an inhibitor on gram-negative bacteria, 50 μg/ml) and vancomycin (an inhibitor on gram-positive bacteria, 50 μg/ml) would inhibit or kill most intestinal bacteria [28]. However, the treatment only slightly increased the SUA (P>0.05, versus normal group, Table 2), which suggested that the bacteria was a relatively minor factor that affected the SUA. Theoretically, uric acid in urate-ip, adenine-ip, and inosine-ip groups could significantly increase, but the expected results did not appear. On the contrary, adenine and inosine by oral administration significantly increased the SUA (Table 2).

### Uric acid in the intestinal juice

**Fig 1:**
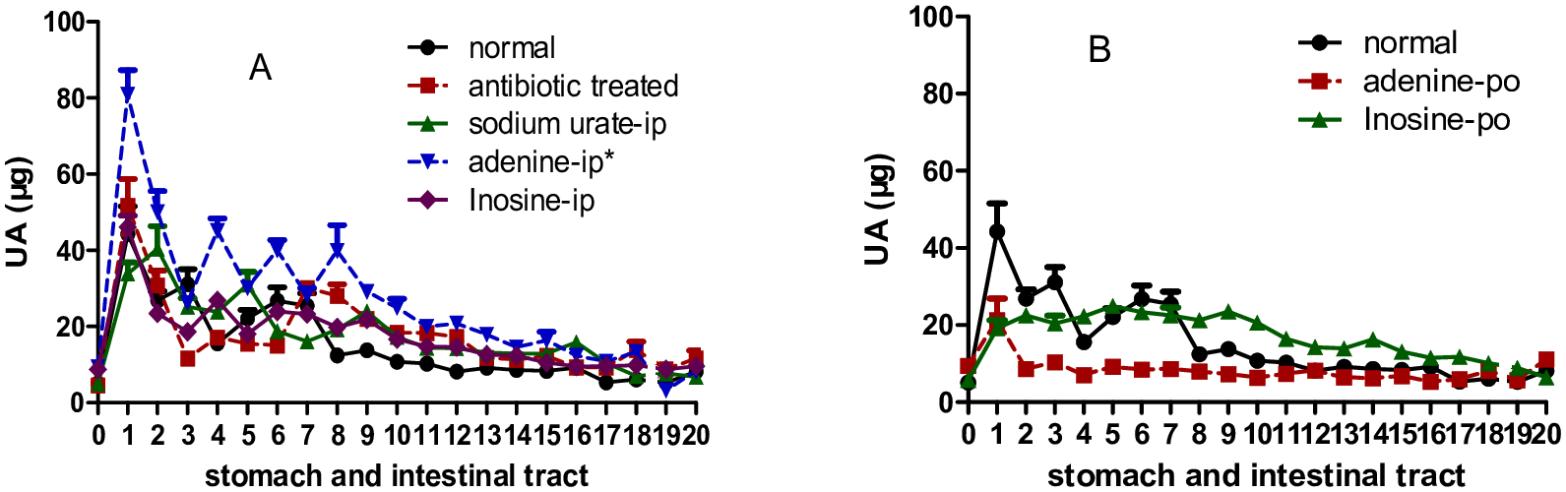
Uric acid distributed in stomach juice and intestinal juice of different segments (mean ± SE, n = 10). Rat intestinal tract except cecum was equally divided to 20 segments, and uric acid in the intestinal juice was assayed. Segment 0 was stomach, segment 1 was duodenum, segment 2 to 7 belonged to jejunum, segment 8 to 18 belonged to ileum, and segment 19 to 20 was colon. In all the groups, the uric acid distribution curve arrived to the maximum at segment 1, and almost declined all the way to the end. Compared with the distribution curve of normal group, those of antibiotic treated, urate-ip, adenine-ip, and inosine-ip groups increased to somewhat extent (A), whereas the curves of adenine-po and inosine-po groups decreased (B). UA, uric acid, * P<0.05 vs normal group, two way ANOVA.

Uric acid in the stomach juice and intestinal juice can be detected. Strangely, in all the groups, uric acid in stomach juice was very low, but high in the intestinal juice especially in the first segment (duodenum). All the uric acid distribution curves in the intestinal juice shared similar features as the curve arrived to the maximum at segment 1, and almost declined all the way till the end (Fig 1). Compared with the distribution curve of normal group, the antibiotic treated, urate-ip, adenine-ip, and inosine-ip group distribution curves were increased to some extent (Fig 1A). Whereas those of adenine-po and inosine-po groups showed a decrease (Fig 1B), which was supported by the results of total uric acid in the intestinal tract (Table 2). As the colon juice contained little uric acid, it was acceptable that the amount of uric acid directly excreted through intestinal tract was unimportant, agreeing with the traditional knoweledge.

### Relationship between uric acid in serum and total uric acid in intestinal juice

**Fig 2:**
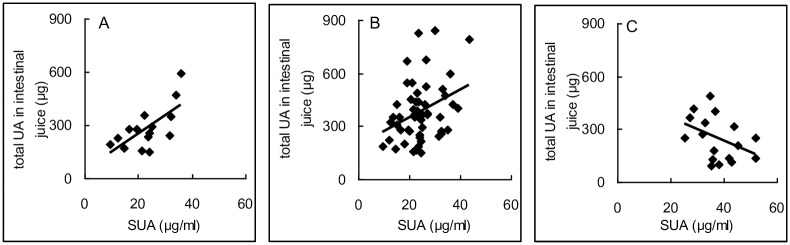
The relationship of uric acid between in serum and intestinal juice. In normal group, there was a good positive correlation between SUA and total uric acid in intestinal juice (A, r = 0.680, P = 0.003, n = 16), and there was also a good positive correlation in antibiotic treated, urate-ip, adenine-ip and inosine-ip groups (B, r = 0.227, P < 0.032, n = 40). However, in adenine-po and inosine-po groups, the correlation became negative though not significantly (C, r = -0.400, P = 0.112, n = 20). Pearson correlation, two-tailed. SUA, serum uric acid; UA, uric acid.

In order to find out the original source of uric acid in the intestinal juice and to explain the possible mechnism, it was necessary to explore the relationship of uric acid between serum and intestinal juice. In normal rats, there was a good positive correlation between uric acid in serum and total uric acid in intestinal juice (r = 0.680, P < 0.05, Fig 2A). Similarly, a good positive correlation but with a smaller correlation coefficient (r = 0.227, P < 0.05 Fig 2B) was also showed in the antibiotic treated and intraperitoneal groups (urate-ip, adenine-ip and inosine-ip groups). However, the correlation became worse in the intragastric groups (adenine-po and inosine-po groups), (r = -0.400, P > 0.05, Fig 2C). The results suggested that adenine and inosine by oral adminsitration were able to increase the SUA, but absolutely or relatively decreased the uric acid distribution in the intestinal juice. This might in turn increase the burden of kidney to excrete uric acid.

Adenine and insosine are precursors of uric acid [2], and their structure (especially adenine) is similar to that of uric acid, which could disturb the transportation of uric acid. Here, it can be deduced that uric acid in the intestinal juice was highly correlated with SUA, and no interferences can be drawn from the intestinal juice that was associated with uric acid synthesis. Since the uric acid in the juice of segment 1 reached to the maximum level, the results demonstrated that the uric acid could come from the liver, pancreas, or directly from the tissue of Segment 1.

### Uric acid source of the intestinal juice

In order to explore the reason of high level of uric acid in the juice of the initial intestinal segment, uric acid in different tissues was assayed (Table 1). According to the results presented in Table 1, the high levels of uric acid in the intestinal juice of segment 1 could be directly associated with the tissues of the segment as the level of uric acid in the tissues was the highest. Segment 1 is the place where bile and pancreatic juice were secreted. Generally, it is possible for uric acid secreted from the bile and pancreatic juice, but the uric acid that is secreted in the juice by them should be at a relatively higher level. The rats in the present study were in a state of fasting, and there could be little bile and pancreatic juice secreted. Unfortunately, compared with the uric acid in the intestinal juice of segment 1 or in its tissue, the level of uric acid in the liver and pancreas was much lower (Table 1). Therefore, the high level of uric acid in the inestinal juice of segment 1 was not likely directly coming from liver and pancreas, but endogenously from the intestinal tissue.

### Bacterial abundance in the small intestinal tract

**Fig 3:**
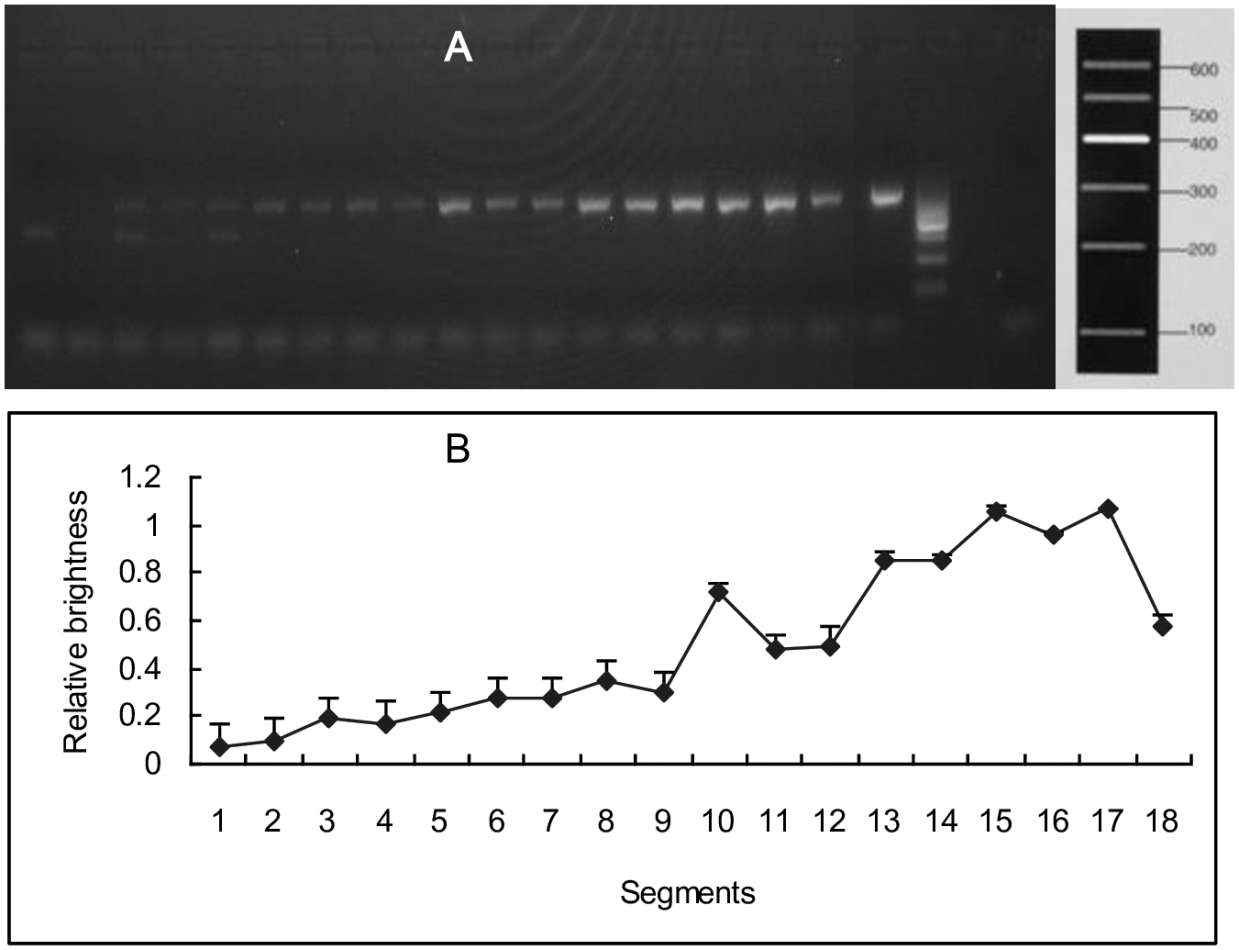
Bacterial abundance in small intestinal tract. Products of 340 bp were amplified with primers Bakt_341F and Bakt_805R (A). From Segment 1 (duodenum) to the end of the small intestinal tract (Segment 18), bands of 16S rRNA became brighter and brighter. However, bands of segment 1 to 7 that was or belonged to duodenum and jejunum was very dark, which suggested there were fewer bacteria inside the intestinal tract. The bands from left to right was segment 1 to 18, positive control, marker, and negative control. The brightness of the bands were obtained and the relative brightness (B, mean ± SD, n = 3) was calculated by the formula, relative brightness = (sample—background)/control.

Bacteria expressed UOX to degrade uric acid [29]. In order to valuate the effect of bacteria, bacterial abundance in small intestinal tract was investigated. Products of 340 bp from 16S rRNA were amplified with primers Bakt_341F and Bakt_805R. From tissue of segments 1 (duodenum) to 18, bands of 16S rRNA became brighter and brighter suggesting that there were more bacteria inside the latter intestinal tracts. However, bands of Segments 1 to 7 that belonged to the jejunum appeared very dark, suggesting that there were few bacteria inside the intestinal tract (Fig 3). In the 18 segments, the abundance of fungi (18S rRNA) was too low to be detected.

### Expression of genes likely associated with uric acid metabolism and transportation in the upper intestinal tissue and the lower

The results of mRNA sequencing showed that there were 32,662 mRNAs sequenced, among which, 5,355 were expressed significantly different. According to the genes directly associated with uric acid secretion or reabsorption in the kidney, 13 genes were selected [1] (Table 3). Only 5 genes that were significantly and differentially expressed between the upper (duodenum) and the lower (the last 5 cm of ileum) intestinal tissue, among which, Lgals9 (UAT), was the only gene associated with urate transport whose up- regulation reached statistical significance in the upper intestinal tissues. Obviously, the results were not enough to explain the high levels of uric acid in the upper intestinal tract trough urate transportation. As for the uric acid reabsorption, there were no genes that expressed significantly. XDH, a key enzyme in the synthesis of uric acid was up-regulated about 4 times in the upper intestinal tissue (another indirect enzyme, adenosine deaminase (Dad) was also up-regulated 6 times, Table 3), which suggested that the upper intestinal tissue synthesized more uric acid than the lower. Though UOX, the key enzyme for uric acid degradation in rats, was insignificantly up-regulated about 6 times in the lower, the constitutive expression was too low to explain the lower levels of uric acid in the lower intestinal juice.

**Table 3:** Genes associated with uric acid transport or metabolism expressed in intestinal tissues (mean, n = 3).

No.	Gene	FPKM^a^			P	Fold (b/c)	log2 Fold	Alias	Note*
		upper^b^	lower^c^	kidney					
1	Abcg2^d^	91.47	222.85	71.19	0.000	0.41	-1.28	BCRP1	secretion
2	Abcc4	1.21	2.39	17.17	0.000	0.51	-0.98	MRP4	secretion
3	Lgals9	491.29	354.93	13.62	0.039	1.38	0.47	UAT	secretion
4	Slc17a1	0.05	0.00	44.01	1.000	N/A	N/A	NPT1	secretion
5	Slc22a6	0.00	0.01	541.93	1.000	0.00	N/A	OA1	secretion
6	Slc2a9	19.37	23.98	7.21	0.078	0.81	-0.31	GLUT9	reabsorption
7	Slc2a6	1.87	1.49	1.03	0.608	1.26	0.33	GLUT9	reabsorption
8	Slc22a13	0.03	0.02	11.67	1.000	1.50	0.58	OAT10	reabsorption
9	Slc22a8	0.01	0.02	395.78	1.000	0.50	-1.00	OAT3	reabsorption
10	Slc22a12	0.00	0.00	396.99	1.000	N/A	N/A	URAT1	reabsorption
11	Xdh	295.02	78.69	35.45	0.000	3.75	1.91	XOR	synthesis
12	Uox	0.01	0.06	0.03	1.000	0.17	-2.58	UOX	degradation
13	Dad	1461.85	230.33	9.59	0.004	6.35	2.67	DAD	synthesis

* The notes of the former 10 was based on the known information from kidney; N/A, the value was incalculable; FPKM, Fragment Per Kilobases of exon model per Million mapped reads.

^a^mean of FPKM, raw data can be seen in S1 table.

^b^Duodenum.

^c^Segment 18, the end of ileum.

^d^The down-regulation of ABCG2 which secretes urate in the upper intestinal tissue is unsuitable to explain the high level of uric acid in the upper intestinal juice.

XDH and ADA are important enzymes to produce uric acid, and UOX is the only enzyme to degrade uric acid in rat. According to the results showed in Table 4, the highest level of XDH and ADA was expressed in the upper intestinal tissue, agreeing with the highest level of uric acid. However, the low level of UOX was expressed in the tissue of stomach, upper intestine, lower intestine, kidney and other organs. There was a roughly possitive correlation between uric acid levels and XDH levels in the tissues, suggesting that intestinal tissues, especially the upper, are mainly responsible for generating uric acid from endogenous purines.

**Table 4:** Xanthine dehydrogenase (XDH), adenosine deaminase (ADA) and urate oxidase (UOX) expressed at mRNA level in rat tissues detected by mRNA sequencing (mean ± SD, n = 3).

No.	Tissues	FPKM^d^			Uric acid in tissue (μg/mg protein)^c^
		XDH	ADA	UOX	
1	Stomach	34.37±4.01	4.68±1.10	0.08±0.07	8.92±2.16
2	upper intestine ^a^	289.86±43.25	1461.85±337.99	0.01±0.02	18.02±4.50
3	lower intestine ^b^	77.93±13.15	230.33±78.40	0.06±0.05	12.80±3.61
4	kidney	18.76±6.03	9.59±2.00	0.03±0.05	2.81±0.04
5	liver	32.15±1.67	3.15±0.61	382.15±77.79	5.02±0.32
6	brain	2.57±0.19	1.62±0.85	0.14±0.15	1.80±1.14
7	lung	66.10±11.01	19.59±4.84	0.09±0.08	8.51±3.95
8	heart	26.76±13.90	7.83±0.17	0.00±0.00	1.82±0.30

FPKM, Fragment Per Kilobases of exon model per Million mapped reads.

^a^Segment 1 (Duodenum).

^b^Segment 18.

^c^Refer to Table 1.

^d^FPKM of XDH, ADA and UOX in the upper intestinal tissue and the lower referred to Table 3.

In liver, both XDH and UOX were expressed at a relatively high level, but the level of uric acid in the tissue was relatively low (Table 4). These results suggested that liver is a door-keeper to catalyze absorbable substances from intestine tract including uric acid and other exogenous purines. Therefore, liver is the main organ to metabolize exogenous purines rather than endogenous ones.

**Fig 4:**
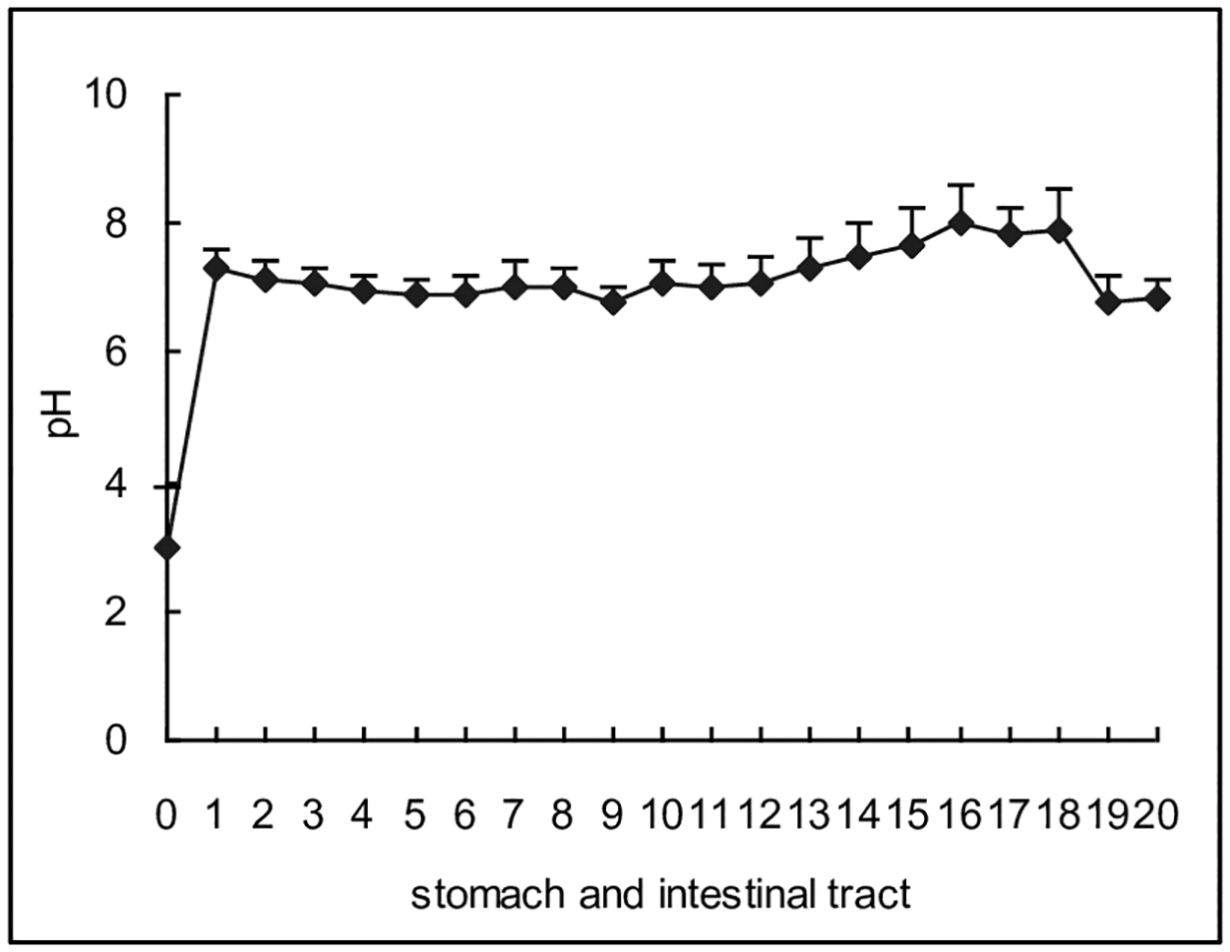
pH of stomach juice and intestinal juice in different segments (mean ± SD, n = 10). Rat intestinal tract except cecum was equally divided to 20 segments, and pH of stomach and intestinal juice was assayed. Segment 0 was stomach, segment 1 to 7 belonged to jejunum (segment 1 was duodenum), segment 8 to 18 belonged to ileum, and segment 19 to 20 was colon. At first, pH in stomach juice was at a low level, and quickly arrived to a relative high level in the juice of segment 1. Then, pH kept at the high level till the end of ileum juice. Finally, pH slightly decreased in the colon juice.

Uric acid is a weak acid with small molecular weight (MW = 168.1). The molecule is permeable via biomembranes and can be transported faster via transpoters. Therefore, uric acid was preferred in moving into the alkaline environment. The main alkaline substance in the body is bicarbonate (HCO_3_^-^), and the substance can be secreted or reclaimed by many proteins including exchangers, ion channels, and some enzymes, the original energy of which may occur from Na^+^/K^+^ ATPase. According to the results of mRNA sequencing, there were 44 transporters, exchangers or enzymes associated with bicarbonate movement, 23 of which (Table 5) were expressed significantly and differently between the upper and lower intestinal tissues. Among these genes, 18 were up-regulated in the upper, the action of which was enough to neutralize the gastric acid and maintain alkalinity in the upper intestinal juice. Actually, as a result of proteins, intestinal juice in the upper intestinal tract (segment 1) was alkaline and the alkaline environment was maintained till the end of ileum (Fig 4).

**Table 5:** Gene products associated with alkaline substance movement expressed between intestinal tissues with significance (mean, n = 3).

No.	Gene	FPKM		P	Fold (b/c)	log2 Fold	Notes^a^
		upper^b^	lower^c^				
1	Atp1a2	1.662	6.332	0.000	0.262	-1.930	Na^+^/K^+^ ATPase, alpha subunit; Salivary secretion, Gastric acid secretion, Pancreatic secretion, Bile secretion, Proximal tubule bicarbonate reclamation
2	Car2	203.305	6.638	0.000	30.627	4.937	Carbonic anhydrase; Gastric acid secretion, Pancreatic secretion, Bile secretion, Proximal tubule bicarbonate reclamation, Collecting duct acid secretion
3	Car4	10.745	55.439	0.000	0.194	-2.367	Carbonic anhydrase; carbonate dehydratase activity, bicarbonate transport; Proximal tubule bicarbonate reclamation
4	Cftr	16.078	9.347	0.000	1.720	0.783	Multidrug resistance-associated protein / mitoxantroneresistance protein; bicarbonate transport; Gastric acid secretion, Pancreatic secretion, Bile secretion
5	Gls2	5.972	1.913	0.000	3.122	1.642	Glutaminase; Proximal tubule bicarbonate reclamation
6	Mdh1	596.550	219.011	0.000	2.724	1.446	Malate dehydrogenase; Proximal tubule bicarbonate reclamation
7	Slc26a2	12.436	22.130	0.000	0.562	-0.831	Sulfate/bicarbonate/oxalate exchanger SAT-1 and related transporters
8	Slc26a6	139.624	43.011	0.000	3.246	1.699	
9	Slc26a8	3.765	1.759	0.000	2.140	1.098	Sulfate/bicarbonate/oxalate exchanger SAT-1 and related transporters; bicarbonate transmembrane transporter activity
10	Slc4a1	0.913	0.095	0.000	9.611	3.265	Na^+^-independent Cl/HCO_3_ exchanger AE1 and related transporters; bicarbonate transport; Collecting duct acid secretion
11	Slc4a10	2.293	0.119	0.000	19.269	4.268	Na^+^-independent Cl/HCO_3_ exchanger AE1 and related transporters
12	Slc4a7	134.153	5.049	0.000	26.570	4.732	
13	Slc9a3	33.400	4.254	0.000	7.851	2.973	Sodium/hydrogen exchanger protein; Bile secretion, Proximal tubule bicarbonate reclamation
14	Aqp1	78.772	130.902	0.001	0.602	-0.733	Aquaporin; Bile secretion, Proximal tubule bicarbonate reclamation
15	Slc25a10	271.059	163.279	0.001	1.660	0.731	Mitochondrial oxoglutarate/malate carrier proteins; Proximal tubule bicarbonate reclamation
16	Slc38a3	2.760	0.073	0.001	37.808	5.241	Proximal tubule bicarbonate reclamation
17	Atp1b3	48.676	33.462	0.002	1.455	0.541	Na^+^/K^+^ ATPase, beta subunit; Salivary secretion, Gastric acid secretion, Pancreatic secretion,Bile secretion, Proximal tubule bicarbonate reclamation
18	Slc26a3	154.492	103.963	0.005	1.486	0.571	Sulfate/bicarbonate/oxalate exchanger SAT-1 and related transporters; Pancreatic secretion
19	Glud1	256.537	173.367	0.014	1.480	0.565	Glutamate/leucine/phenylalanine/valine dehydrogenases; Proximal tubule bicarbonate reclamation
20	Pck1	46.480	64.463	0.024	0.721	-0.472	Phosphoenolpyruvate carboxykinase; Proximal tubule bicarbonate reclamation
21	Pck2	18.965	14.447	0.026	1.313	0.393	Phosphoenolpyruvate carboxykinase; Proximal tubule bicarbonate reclamation
22	Slc4a4	33.257	25.382	0.028	1.310	0.390	Na^+^-independent Cl/HCO_3_ exchanger AE1 and related transporters; Pancreatic secretion, Bile secretion, Proximal tubule bicarbonate reclamation
23	Slc26a11	3.747	2.459	0.031	1.524	0.608	Sulfate/bicarbonate/oxalate exchanger SAT-1 and related transporters

FPKM, Fragment Per Kilobases of exon model per Million mapped reads; N/A, the value was incalculable.

^a^The note was based on the known information from KOG (euKaryotic Orthologous Groups database), GO (Gene Ontology database) and KEGG (Kyoto Encyclopedia of Genes and Genomes pathway enrichment analysis).

^b^Duodenum.

^c^Segment 18, the end of ileum.

### XDH and UOX at protein level in intestinal tract

**Fig 5:**
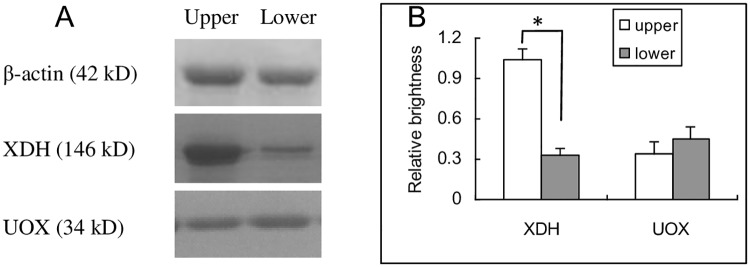
XDH and UOX expressed at protein level in the upper and the lower intestinal tissues. XDH in the upper tissue was obviously up-regulated at protein level, but UOX in the lower was slightly up-regulated (A). The brightness of the bands were obtained and the relative brightness (B, mean ± SD, n = 3) was calculated by the formula, relative brightness = (sample—background)/β-actin. * P<0.05, student t test. Upper, duodenum; Lower, Segment 18, the end of ileum; XDH, xanthine dehydrogenase; UOX, urate oxidase.

The results of mRNA sequcing suggested that the up-regulation of XDH was likely to be the reason for high level of uric acid in duodenum tissue. The Western blot results showed that expression of XDH was up-regulated at protein level (Fig 5). However, the expression of UOX was only slightly changed at a low level (Fig 5).

### SUA decreased by oral UOX in rats

**Fig 6:**
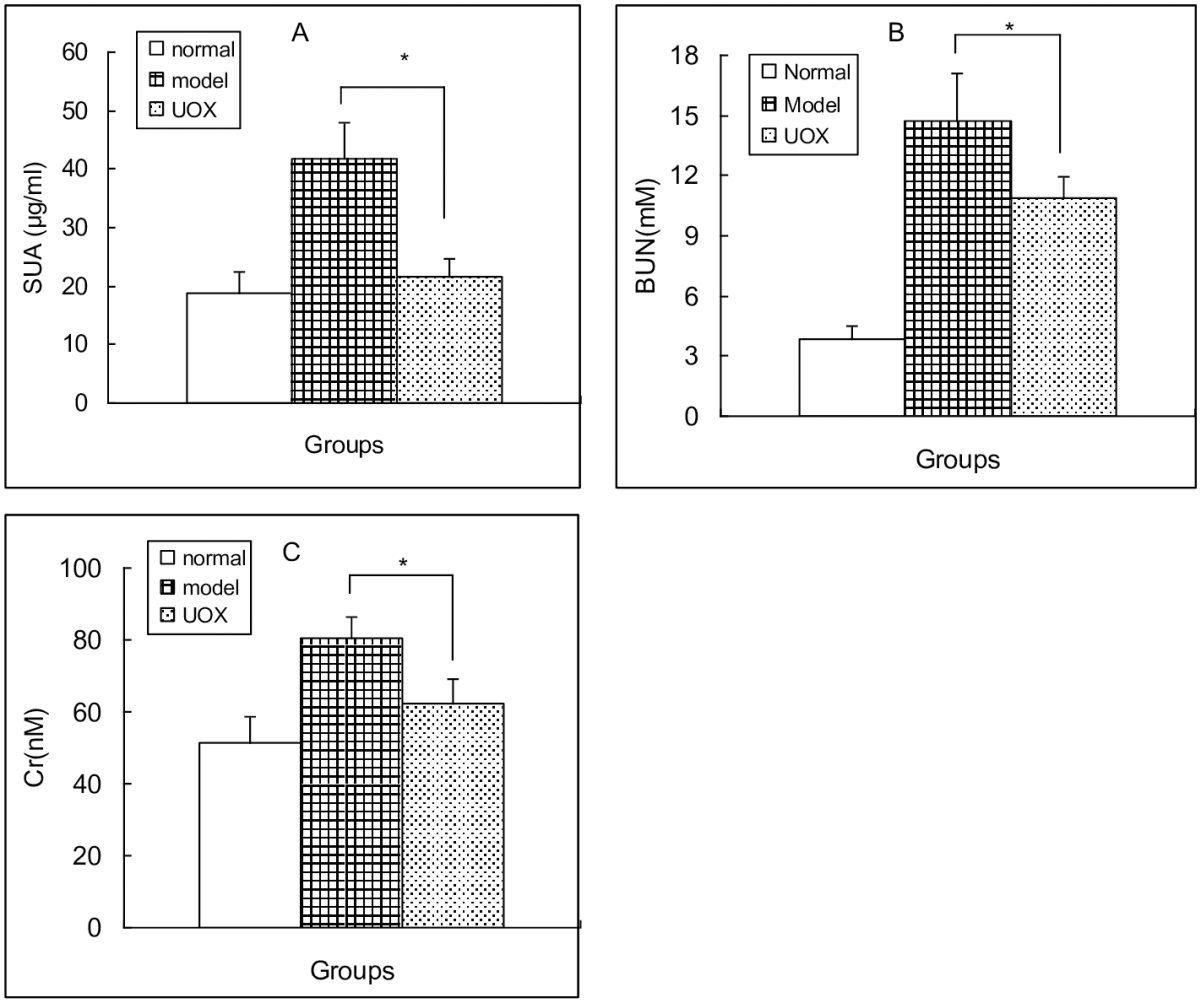
Oral UOX decreased SUA (A), BUN (B) and Cr (C) (mean ± SD, n = 10). Rats in model group were gavaged with Mixture A of 10 ml/kg in the morning and with Mixture B of 5 ml/kg in the afternoon. Rats in UOX group were gavaged with Mixture A of 10 ml/kg in the morning and with Mixture B of 5 ml/kg of the same volume but added UOX of 4 u/ml in the afternoon. Rats in normal group were gavaged with normal saline of the same volume both in the morning and the afternoon. Administration was taken for 5 days. Mixture A containing adenine (15 mg/ml), potassium oxonate (15 mg/ml), omeprazole (0.4 mg/ml) and CMC-Na (0.5%); Mixture B containing NaHCO_3_ (12 mg/ml) and horse serum (50%); * P < 0.05, UOX group vs model group; SUA, serum uric acid; BUN, blood urea nitrogen; Cr, blood creatinine.

In model group, oral administration of adenine (150 mg/kg) and potassium oxonate (150 mg/kg) in rats was able to increase SUA (Fig 6A), BUN (Fig 6B) and Cr (Fig 6C), suggesting that the combination of adenine and potassium oxonate was able to cause hyperuricemia and renal function damage. In UOX group, oral UOX decreased all of them (Fig 6).

## Discussion

CKD has a serious influence to human health. CKD is characterized by the loss of renal cells and accumulated extracellular matrix and injured renal function led to metabolic disorder [30,31,32,33,34]. Unsoluble uric acid and othe purines with low solubility will cause renal tubules obstruction and activate redox pathway and inflammatory pathway which lead to renal fibrosis and renal dysfucntion [35,36,37,38,39,40]. In turn, renal dysfunction will deteriorate uric acid excretion. Therefore, lowering SUA by inhibiting syntheis or enhancing excretion through kidney or othe organs is a key treatment to solve the CKD related problems.

### Intestinal tract should be a very important organ for uric acid distribution and a potential target for uric acid removal

Until recently, most studies ignored the importance of intestinal tract for uric acid distribution. At first, the present study demonstrated that the existence of uric acid in the intestinal juice, especially in segment 1. In normal rats, the total uric acid in the intestinal tract was 2 times more than that in the serum. The total uric acid in the intestinal tract was still more than that in the serum even rats treated with factors associated with uric acid synthesis. Therefore, the good correlations of uric acid between the intestinal juice and serum can be established. The results suggeted that, if all the uric acid in the intestinal juice was removed or degraded, it was equivalent to that in the serum cleared once or twice. Of course, the present study layed the foundation to the inspiring strategy of lowering SUA through intestinal tract, and could explain some treatment (montmorillonite) that was able to decrease SUA through intestinal tract [17], and give a new clue to explain that some natural product (mangiferin) [41] with low bioavailability was able to lower the SUA.

The present study did not deny the previous opinion that uric acid excreted through intestinal tract was about one third. Our results showed that little uric acid was distributed in the colon, and the uric acid excreted by this way was a very small fraction. However, there is still one question as to what mechanism was involved for the distribution of uric acid profile in the intestinal tract.

### Mechanism for the high level of uric acid in the upper intestinal juice

The high levels of uric acid in the juice of segment 1 could not come from stomach (because uric acid in stomach juice was too low), but could be from liver or pancreas where their secretory fluid can be dumped into. Liver is the main organ that transforms the exogenous and endogenous substances, and could be one of the main producer of uric acid [9]. If the high levels of uric acid come from the bile or pancreatic juice, uric acid inside the intestinal segment 1 could be removed easily just by rinsing, and the uric acid in the intestinal tissue could be low. However, our results showed that uric acid both in the liver and pancreatic tissues was lower than that in the intestinal tissue of segment 1 even after the inner wall was rinsed. Therefore, the high level of uric acid in segment 1 was less likely to come from the liver and pancreas, but more likely from the intestinal tissues of segment 1.

Though the present study provided solid evidence that high level of uric acid was from the intestinal tissues, it is neccessary to explain the underlying mechanism. There were three possibilities: the high level of uric acid was caused by over-synthesis, over-secretion, or both. According to the results of mRNA sequencing, the uric acid producer XDH expressed in segment 1 was at a higher level, and as much as about 4 times that of segment 18, which was further confirmed by the results of Western blot. Similar results were observed in human biopsy [9]. As for the uric acid destroyer, UOX, its expression was not significant between segment 1 and segment 18 both at the mRNA and protein levels. However, the high level of uric acid in segment 1 could not fully be explained by uric acid synthesis, since there were no reasons as to why the uric acid over-synthezied by XDH must be secreted to the intestinal tract, and it was also possible for uric acid to be diffused to blood and carried away.

Nevertheless, the level of uric acid in the serum was much lower than that in the intestinal juice of segment 1, and most uric acid was synthesised by XDH, which could be excreted into the intestinal tract. In the resting state, intestinal mucosa secreted enough alkaline juice to neutralize the gastric acid and maintained a weak alkaline enviroment in the small intestine. The main alkaline juice was secreted by the mucosa of segment 1, because the intestinal juice quickly turned into alkaline in the segment. Actually, most proteins associated with alkaline juice secretion in segment 1 were then up-regulated compared to those in segment 18. Therefore, as a weak acid, it was likely for uric acid to be diffused into segment 1 accompanying the bicarbonate secretion.

Therefore, both up-regulated XDH and alkaline juice were the main factors that keep up the high levels of uric acid in the juice of segment 1.

### Mechanism for the low level of uric acid in the lower intestinal juice

The distribution of uric acid was lower in the lower intestinal tract. This phenomenon was caused either by uric acid degradation [42] or by uric acid reclamation [17]. There were many bacteria in the intestinal tract (but fungi were undetectable in the present study) that were able to degrade uric acid. Especially, the pattern of bacterial distribution coincided with that of uric acid distribution. Based on the known knowledge, it was possible to explain that the low levels of uric acid in the lower intestinal tract was caused by bacteria [42]. However, rats treated with gentamicin and vancomycin were not able to fully prove the decrease because the uric acid distribution curve was also similar to that of the normal group. Therefore, bacteria were not considered as the main factor to explain the low levels of uric acid, and the phenomenon could be caused by uric acid reclamation in the subsequent intestinal tract. And actually, uric acid was absorbable, because hyperuricemia model was able to be established by oral intake of uric acid [43] both in mice [44] and quails [45], and some transporter associated like ABCG2 was found in intestinal tract [3].

Therefore, the uric acid distribution could be explained as follows. Excessive uric acid in the tissues of initial intestinal tract is synthesized by XDH, and may enter the intestinal tract by diffusion, augmented by pH-trapping in the alkaline environment of the upper intestinal tract, due to the high concentration of bicarbonate. This causes high level of uric acid in the initial intestinal juice. Afterwards, uric acid was dominantly reclaimed slowly into the blood which can be metabolized to allantoin by UOX in rat’s liver, and a small part of it was degraded by bacteria, which in turn caused the low level of uric acid in the subsequent lower tract.

Finally, the high level of uric acid in upper intestinal juice was proved by oral UOX in an animal model. In the present study, omeprazole (an inhibitor on gastric acid) was introduced to protect UOX from beeing destroyed by gastric acid and pepsin [46]. Also, horse serum was included in order to protect oral UOX from proteolytic destruction. With their protection, UOX passed through stomach and safely arrived at the upper intestinal tract to degrade uric acid. Adenine with potassium oxonate increased SUA and caused renal function damage. Unsurprisingly, not only did oral UOX decrease SUA, but also improved renal function in the model.

## Conclusions

The present study established that the intestinal tract was both a very impotant place for uric acid distribution and a target organ for uric acid removal. This finding could open a new window for studying the uric acid excretion and provide new clues for SUA lowering.

## Supporting information

S1 Table FPKM of gene directly associated with uric acid transportation and metabolism expressed at mRNA level in stomach, small intestinal tissue and kidney (mean ± SD, n = 3). (DOC)
