# Supplementary material for: Intestinal tract is an important organ for lowering serum uric acid in rats
Source: PLoS One. 2017 Dec 21;12(12):e0190194. doi: 10.1371/journal.pone.0190194 (PMC5739491; doi:10.1371/journal.pone.0190194)
Supplement: S1 Table — (DOC) [file pone.0190194.s001.doc]

FPKM of gene directly associated with uric acid transportation and metabolism expressed at mRNA level in stomach, small intestinal tissue and kidney (mean ± SD, n=3)

| Gene | stomach | ^a^upper | ^b^lower | kidney | note |
| --- | --- | --- | --- | --- | --- |
| Abcg2 | 0.26±0.13 | 70.44±12.98 | 210.22±5.10 | 71.19±10.03 | secretion |
| Abcc4 | 7.43±2.40 | 1.13±0.32 | 2.74±0.43 | 17.17±2.02 | secretion |
| Lgals9 | 259.23±179.61 | 434.31±94.21 | 397.13±40.59 | 13.62±2.34 | secretion |
| Slc17a1 | 0±0 | 0±0 | 0±0 | 44.01±4.75 | secretion |
| Slc22a6 | 0±0 | 0±0 | 0.01±0.02 | 541.93±36.04 | secretion |
| Slc2a9 | 0.58±0.104 | 15.28±2.56 | 24.23±7.20 | 7.21±0.36 | reabsorption |
| Slc2a6 | 2.45±0.32 | 1.70±0.30 | 1.61±0.26 | 1.03±0.06 | reabsorption |
| Slc22a13 | 0.02±0.03 | 0.14±0.06 | 0.22±0.20 | 11.67±3.25 | reabsorption |
| Slc22a8 | 0.03±0.06 | 0±0 | 0.03±0.02 | 395.78±47.31 | reabsorption |
| Slc22a12 | 0.01±0.02 | 0±0 | 0.14±0.13 | 396.99±33.77 | reabsorption |
| Xdh | 18.94±5.88 | 261.88±15.59 | 89.74±14.30 | 35.45±3.79 | synthesis |
| Uox | 0.08±0.07 | 0.02±0.03 | 0.08±0.07 | 0.03±0.06 | degradation |
| Ada | 4.68±1.10 | 1461.85±337.99 | 230.33±78.40 | 9.59±2.00 | synthesis |

a, the initial segment of small intestinal tract.

b, the end segment of small intestinal tract.
